# Inter-relatedness of underlying factors for injury and violence among adolescents in rural coastal Kenya: A qualitative study

**DOI:** 10.1177/2055102919849399

**Published:** 2019-05-13

**Authors:** Derrick Ssewanyana, Anneloes van Baar, Patrick N Mwangala, Charles R Newton, Amina Abubakar

**Affiliations:** 1 Kenya Medical Research Institute (KEMRI), Kenya; 2 Utrecht University, The Netherlands; 3 Pwani University, Kenya; 4 University of Oxford, UK; 5 Aga Khan University, Kenya

**Keywords:** adolescents, injury, socio-ecological, violence

## Abstract

We utilized a socio-ecological model to explore views from 85 young people and 10 local stakeholders on forms and underlying factors for unintentional injury, violence, self-harm, and suicidal behavior of adolescents in Kilifi County, Kenya. Young people took part in 11 focus group discussions, whereas 10 in-depth interviews were conducted with the local stakeholders. Road traffic accidents, falls, fights, sexual and gender-based violence, theft, and vandalism were viewed as common. There was an overlap of risk factors, especially at intra- and interpersonal levels (gender, poverty, substance use, parenting behavior, school drop-out). Some broader-level risk factors were insecure neighborhoods and risky sources of livelihood. Research is needed to quantify burden and to pilot feasible injury prevention interventions in this setting.

## Introduction

The need to address injury and violence has been prioritized by the sustainable development goals (SDG) of the United Nations, specifically goals 3 and 16 (UN-DESA, 2015). Violence is the use (either actual or by threat) of physical force or power against someone, a community, or a group, and it has the likelihood of resulting in psychological harm, injury, mal-development, deprivation, or death (World Health Organization (WHO), 2014). Injuries account for 10 percent of the global burden of disease and caused 4.8 million deaths in 2013. The majority of such deaths resulted from road traffic injuries (29%), self-harm (18%), falls (12%), and interpersonal violence (9%) (Haagsma et al., 2016). By 2030, road traffic injuries will contribute the third largest burden of disease globally (Mathers and Loncar, 2006).

Adolescents are vulnerable to violence and injury (Johnson and Jones, 2010). Their vulnerability may arise from the challenges they face in coping to rapid developmental changes, impulsiveness, their increased need for self-agency, and social and economic deprivation (WHO, 2009). The burden among adolescents is possibly underestimated because victims of non-fatal forms of violence and injury (sexual, physical, and psychological) may often not seek services at health facility level (WHO, 2014). Nonetheless, sub-Saharan Africa (SSA) is the most disproportionately impacted region by burden of injury and violence (Haagsma et al., 2016) which underscores an urgent need to understand the dynamics surrounding this burden among adolescents and to develop interventions in this region. As an example, disability-adjusted life years (DALY) for road injuries among boys and girls is nine times higher in SSA than that in high-income Asia Pacific (Haagsma et al., 2016). Focusing on Eastern SSA, road injuries, drowning, falls (in both gender), and self-harm among males are among the top 15 causes of mortality in younger adolescents (10–14 years) (Kassebaum et al., 2017). In this region, among the older adolescent age group (15–19), road injuries and interpersonal violence (among both genders) and self-harm, collective violence, and drowning (only among males) are among the top 15 causes of mortality (Kassebaum et al., 2017).

Kenya is one of the countries in Eastern SSA where a high burden of injury and violence has been documented among adolescents (Botchey et al., 2017; Brown et al., 2008; McKinnon et al., 2016; United Nations Children’s Fund (UNICEF), 2012). However, the underlying factors so far are not well documented. In some Kenyan health facilities, about a half of the casualties are youths (15–29 years) presenting with injuries from road traffic accidents (37%), falls (26%), and physical assault (20.1%) (Botchey et al., 2017). In 2010, about 48 percent of Kenyan adolescents (13–17 years) had experienced some form of physical violence and 11 percent of females and 4 percent of male adolescents had experienced some form of sexual violence within the preceding 12 months (UNICEF, 2012). Noteworthily, there was an overlap in the accounts of physical, sexual, and emotional violence (UNICEF, 2012). Another study reported the occurrence of bullying among adolescents from Kenya as the third highest (almost 60%) among eight African countries of Kenya, Morocco, Namibia, Swaziland, Tanzania, Uganda, Zambia, and Zimbabwe (Brown et al., 2008).

The underlying risk factors for adolescent injury and violence may be context specific and yet rarely comprehensively reported in some settings such as SSA. Evidence from Europe and North America points to variation in causes of fatal and non-fatal injuries, with most of the fatal injuries being linked to road traffic accidents, drowning, poisoning, falls, and burns, whereas most non-fatal ones often result from fights and physical activity or sports (Molcho et al., 2015). Across 30 countries from Europe and North America, notable risk factors for injury and violence include gender (higher injury rates among males), psychoactive substance use, more frequent physical activity and sports, and higher levels of family affluence (Molcho et al., 2015). Variations in the occurrence of injury and violence during adolescence across countries in the African context have also been reported (Peltzer, 2008). A multi-country study on injury and violence among adolescents from six African countries showed that the majority of such injuries arise from physical activities and sports, falls, motor and road traffic accidents, fights, assault, and self-infliction. The significant underlying risk factors for injury were gender (higher in males), lower age, truancy, drug use, low socio-economic status, depression, and engagement in multiple forms of risk behavior (Peltzer, 2008). Some documented underlying factors for violence and injury among adolescents in the Kenyan context include insecurity, sub-optimal mental health, poor parent–child relationship, and social media (UNICEF, 2012). Being male and experience of adverse life events have also been linked to adolescent delinquent behavior like weapon carrying, theft, and assault in Kenya (Kabiru et al., 2014; McKinnon et al., 2016).

There are however indications of regional disparities by forms and burden of injury and violence among adolescents in Kenya (Marsha, 2015; National Council for Population and Development (NCPD), 2017). It is plausible that certain unique risk factors explain the existing disparities in burden of violence and injury among adolescents in the Kenyan context. Understanding context-specific risk factors can facilitate the design of targeted and effective interventions to address this problem. Within Kenya, Kilifi County is among the disproportionately impacted regions where, for example, a high burden of sexual and gender-based violence is reported (Marsha, 2015; NCPD, 2017; Ssewanyana et al., 2017). Much as 22 percent of the residents in Kilifi are adolescents (Kenya National Bureau of Statistics (KNBS), 2009), the evidence is still scarce on the forms of injury and violence experienced by adolescents in Kilifi County and the underlying factors are also still inadequately explored. There have been a few studies that report weaknesses in the judicial system, cultural practices, parental negligence, insecurity, poverty, unemployment, and land disputes as some significant predisposing factors (Kesho-Kenya, 2016; NCPD, 2017; Ssewanyana et al., 2017). Given that many non-fatal and less serious injuries commonly experienced during adolescence are rarely reported at health facilities and that detailed reports on their underlying causes are often not well documented at health facilities (Botchey et al., 2017), it is insufficient to only rely on health facility data for research and intervention planning. Furthermore, rural settings such as Kilifi are often characterized by low healthcare and social service coverage, emerging risks for young people due to rapid urbanization, and entrenched poverty among households (NCPD, 2017; Scott et al., 2012). Such conditions may heighten vulnerability to injury and its consequences during adolescence. Thus, there is urgent need for more rigorous qualitative research that actively engages young people and other community members in generating more enriched understanding of common forms of violence and injury, their explanatory factors, and how these factors are experienced and construed by young people within Kilifi County. Understanding these specific forms, factors, and processes is fundamental for policy and intervention planning.

Ecological models have been recommended for analyzing underlying factors and planning for interventions for violence and injury prevention (Allegrante et al., 2010, Centers for Disease Control and Prevention (CDC), 2017), but no studies originating from Kilifi County utilize such models. The socio-ecological model (McLeroy et al., 1988) is increasingly applied in injury and violence research and prevention programs (Baron-Epel and Ivancovsky, 2015; Bogardus et al., 2019; Gashaw et al., 2018). The socio-ecological model builds from earlier theories of Bronfenbrenner (1977) that explain human development as being a product of interactions from multi-person systems and environmental factors beyond the immediate individual circumstances. The socio-ecological model defines five levels of interaction that determine human behavior: intrapersonal-level factors (such as income, education, and attitudes); interpersonal-level factors (such as social networks, family, and friendships); institutional-level factors (such as organizational characteristics and regulations); community-level factors (e.g. relationships among informal networks, institutions, and organizations); and public policy–level factors which consist of local and national laws or policies. Utilizing a socio-ecological approach (McLeroy et al., 1988) to explore the views of young people and local stakeholders in Kilifi County at the Kenyan coast, this study intends to contribute toward a clearer conceptualization of the prevalent forms of unintentional injury, violence, self-harm, and suicide-related behavior of adolescents and the underlying risk and protective factors.

## Methods

### Study setting and participants

A detailed description of the design and methodology of this study is described elsewhere (Ssewanyana et al., 2017, 2018).

Briefly, this qualitative study was conducted between August and November 2016 in Kilifi County at the coast of Kenya. The study participants were young people and local stakeholders. The young people comprised primary and secondary school students, adolescents who had dropped out of school, and adolescents living with HIV and attending an HIV clinic in Kilifi. Young adults (20–30 years) who served as community representatives also participated because they were presumed to have a good understanding of the concerns of youths in Kilifi. Local stakeholders were adults who worked with institutions and organizations providing services to adolescents in Kilifi such as teachers, clinicians, and employees of community-based organizations (see Table 1).

**Table 1.** Gender and age characteristics of the study participants.

Participants	Gender	Age group
Rural students	17 males and 17 females	9 young adolescents and 25 older adolescents
Peri-urban students	15 males and 13 females	15 young adolescents and 13 older adolescents
Adolescents living with HIV	5 males and 4 females	6 young adolescents and 3 older adolescents
School drop-out adolescents	5 males and 2 females	4 young adolescents and 2 older adolescents
Stakeholders	4 males and 6 females	27–51 years (*M* = 31)
Young adult community representatives	3 males and 4 females	22–28 years (*M* = 25)

Young adolescents are 10–14 years old; older adolescents are 15–19 years old; *M* refers to median age.

We utilized a snowballing technique to recruit local stakeholders. Young adults were purposively drawn from an existing database of 200 community representatives. Upper primary classes (classes 5–8) and lower secondary classes (classes 1–2) from two primary and two secondary schools were utilized to purposively recruit school-attending adolescents. For purposes of this study, Kilifi town was considered a reference peri-urban setting, whereas the areas with limited social services and within 10 or more kilometers from Kilifi town were referred to as rural settings. We purposively sampled adolescents living with HIV from an existing teen club at their HIV clinic. Adolescents who had dropped out of school were recruited from two community health catchment units. All recruitment processes took into consideration a balanced representation of participants according to their gender, age, and residence.

### Ethical consideration

The ethical approval to conduct this study was obtained from the Kenya Medical Research Institute Scientific and Ethics Review Unit (KEMRI/SERU/CGMR-C/0047/3263). We directly sought prior written informed consent from participants that were 18 years or older. For participants who were less than 18 years of age, written informed consent was provided by their parents or legal caregivers. In addition to written consent from their caregivers, the adolescents of ages 13–17 years gave their written assent for their participation in this research. Permission was also sought from the County Director of Education and school head teachers so as to involve school-attending adolescents.

### Data collection

We conducted 11 focus group discussions (FGDs) with 85 adolescents and young adults: each lasting between 75 and 120 minutes and comprising seven to nine participants. The FGDs for school-attending adolescents were gender disaggregated and took place in the schools. FGDs for adolescents living with HIV and young adults were conducted in a private setting at Kenya Medical Research Institute. And 10 key stakeholders were administered key informant interviews (KIIs) lasting 60–90 minutes each and took place in their preferred venues. The discussions and interviews were guided by an FGD and interview guide which covered various forms of adolescent health risk behaviors. The participants were asked an open-ended question about the specific forms of risky behaviors that predisposed adolescents (10–19 years) to unintentional injury, violence, and self-harm and suicide within Kilifi. They were further probed to give specific examples and context of each suggested form of behavior. Therefore, all the documented views of participants in this article were their spontaneous opinions. All the interviews and discussions were conducted in either English or Kiswahili and moderated by a research officer. The participants granted permission for their discussions to be audio recorded and notes taken. We also administered a socio-demographic data capture sheet for demographic information such as gender, religion, residence, age, education level, and institutional affiliation.

### Data analysis

The audio-records were transcribed verbatim and translated into English by a professional team. An initial coding was conducted by the first author (D.S.) and the third author (P.N.M.) who had both extensively read and reflected on the scripts. A thematic analysis approach (Braun and Clarke, 2006) was utilized. We utilized both inductive and theoretical thematic analysis to explore the opinions on forms of unintentional injury, violence, and self-harm and suicide among adolescents in Kilifi. We undertook an inductive approach to code participants’ views surrounding the various forms of injury, violence, and self-harm and suicide behavior that were experienced and/or perpetrated by the adolescents in Kilifi. Such inductive thematic analysis involved coding the data without trying to fit any pre-existing or pre-conceived coding frame. We applied a theoretical thematic analysis guided by the socio-ecological model (McLeroy et al., 1988) to categorize participants’ views into risk and protective factors for the experienced and/or perpetrated forms of behavior by adolescents in Kilifi. The socio-ecological model postulates five levels of interaction that determine behavior, which are intrapersonal factors (biological or personal history); interpersonal factors (formal, social, and support networks); institutional factors (institutions with organizational characteristics); community factors (linkages among organization, informal and institutional networks); and public policy factors (local, state, and national laws or policies). Final stage coding in NVivo 11 software was conducted by D.S. Thereafter, the codes were discussed among the research team to reach consensus. Charting by case and theme was then conducted and finally reviewed among the research team.

## Results

Key informants comprised two county hospital staff, three teachers, four employees of community-based organizations, and a county government staff. Table 1 presents a description of the study participants by gender and age.

### Forms of unintentional injury, violence, and self-harm and suicide

The most frequently discussed forms of unintentional injuries were road traffic accidents, falls, cuts or bruises, and drowning which were considered common in both genders, while there were also indications that genital trauma resulting from abortion and sexual assault is experienced by many adolescent girls. Physical fights, theft, vandalism, and physical bullying were the main forms of violence. During all FGDs and 80 percent of KIIs, the participants perceived that few incidents of self-harm and suicidal behavior happen among adolescents in Kilifi. Specifically, poisoning and attempted drowning were the most mentioned forms of suicidal or self-harm behavior. Further details on forms of injury and violence are summarized in Table 2.

**Table 2.** Forms of unintentional injury, violence, and self-harm and suicidal behavioral outcomes of adolescents in Kilifi discussed by study participants.

Forms of injury and violence	Percentage of FGDs where the form of injury or violence was discussed (*N* = 11)	Percentage of KIIs where the form of injury or violence was discussed (*N* = 10)
Unintentional injuries		
Road traffic accidents	91 (10)	90 (9)
Being a victim of mob justice	9 (1)	10 (1)
Genital injury	73 (8)	40 (4)
Falls (also involving fractures)	82 (9)	70 (7)
Drowning	91 (10)	70 (7)
Cuts and bruises	54 (6)	40 (4)
Snake bites	9 (1)	10 (1)
Burns	0 (0)	10 (1)
Self-harm and suicidal behavior		
Drowning (or attempted)	36 (4)	30 (3)
Poisoning (or attempted)	82 (9)	50 (5)
Hanging self (or attempted)	27 (3)	10 (1)
Self-inflicted body injuries (e.g. burns or cuts)	18 (2)	0 (0)
Violence		
Theft	82 (9)	80 (8)
Perpetration of mob justice	27 (3)	10 (1)
Physical fights	100 (11)	70 (7)
Vandalism (arson, riots, damaging property)	73 (8)	40 (4)
Bullying (physical)	64 (7)	30 (3)
Perpetration of sexual violence or assault	36 (4)	40 (4)
Perpetration of intimate partner violence	9 (1)	20 (2)
Cyber bullying	36 (4)	0 (0)

FGDs: focus group discussions; KIIs: key informant interviews.

### Underlying risk factors for unintentional injury, violence, and self-harm and suicide-related behavior of adolescents

**Figure 1.**
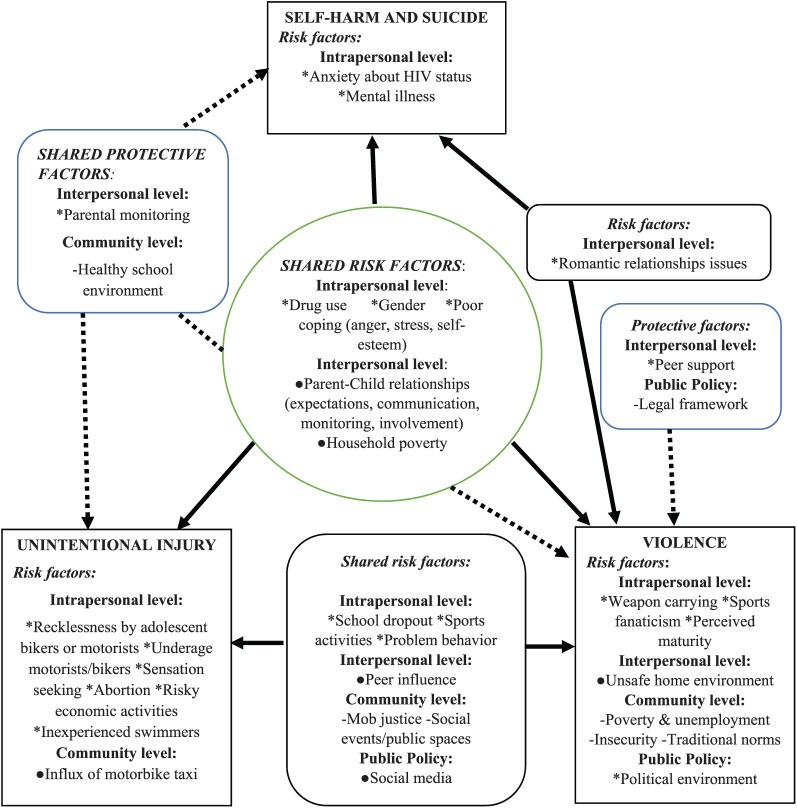
Underlying risk and protective factors for unintentional injury, violence, self-harm, and suicidal behavior of adolescents in Kilifi County.

We found that there were various shared risk factors at intra- and interpersonal levels across all the three categories of violence and injury behavior. However, some risk factors were only shared between two categories. A few other risk factors were behavior specific (see Figure 1).

#### Intrapersonal factors

##### Drug use

The use of alcohol, marijuana, and khat by adolescents was perceived to predispose them to violence, injury, self-harm, and suicide. Some participants argued that many motorbike taxi riders (some being adolescents) and some pedestrians are involved in traffic injuries due to loss of control while being under the influence of drugs. Scenarios of sexual exploitation and physical assault perpetrated by intoxicated adolescents were also shared.

##### Poor coping mechanism

Adolescents’ poor management of daily stressors at home and within the broader community was perceived to increase their susceptibility to violence, injury, self-harm, and suicide. Participants discussed that many adolescents in Kilifi engage in physical violence and mob justice to resolve their conflicts and resentment. Romantic relationship issues such as break-ups and rejection were also recognized as underlying factors, especially for adolescents’ suicidal behavior and fights. Stress, disappointment, anger, low self-esteem, stigma, and social exclusion were other examples of stressors that adolescents grapple with:

For a girl who had sex with a boy and he turns out he was not serious about living with her, she may commit suicide because of bitterness. (Secondary school girl)

##### Gender

There was general consensus that males are more likely than their female counterparts to engage in behavior resulting in unintentional injuries and violence like theft, sexual assault, physical fights, and vandalism. However, from one FGD some young people perceived risk for suicidal behavior to be higher among adolescent girls compared to their male colleagues.

##### School drop-out

School discontinuation was in many ways perceived to predispose adolescents to unintentional injury and violence. Both key informants and young people expressed that school drop-out among male adolescents often results in underage or inexperienced motorbike taxi operation—a practice that they largely linked to traffic accidents. School drop-out was also a reported underlying factor for the initiation of risky habits like drug use and theft owing to loss of behavioral monitoring by school authorities.

##### Sports activities

Typical sports activities like football and athletics as well as potentially high risk sports like skating, swimming competitions in the ocean, taekwondo, and rugby were discussed risk factors for both violence and unintentional injury during a few KIIs and FGDs. These sports activities were linked to common adolescent complaints like cuts, sprains, fractures, drowning, and falls. A few young people also mentioned that sports activities like taekwondo and weight lifting may foster adolescents’ exhibition of violent behaviors.

#### Interpersonal factors

##### Parent (caretaker)-to-child relationship

FGD and KII participants linked unrealistic expectation, harsh communication, and excessive punishment from caretakers (e.g. in the case of an unexpected pregnancy or poor academic performance) to the risk of suicidal ideation by adolescents. Also, the lack of parental involvement and low behavioral monitoring were related to the occurrence of adolescents’ unintentional injuries and violence.

##### Household poverty

Poor housing conditions and lack of basic household necessities like electricity were some factors perceived to predispose adolescents to unintentional injury like in circumstances when houses collapse or in case of fire accidents from cooking or lighting sources. Poor housing conditions were also linked to the risk of insecurity and violence. Besides, it was thought that some adolescents engage in risky economic activities or criminal acts in the search for basic needs and money.

##### Peer pressure and group dynamics

Key informants and young people discussed that peer pressure influences adolescents to perpetrate violence or predisposes some to violence and unintentional injury. For example, some participants reported that certain adolescents may mobilize peers to gang up and commit offenses like robbery, sexual violence, or arson. Others influence their peers to initiate risky activities (e.g. swimming in the ocean). It was also mentioned that some adolescents may get bullied or assaulted because they do not fit in the group.

#### Community level

##### Mob justice

The tendency for young people to take the law in their own hands (mob justice) was discussed in eight FGDs and during four KIIs. Adolescents’ engagement in crimes like theft and being innocent victims of riots were shared examples of how they fall victim to mob justice. It was also pointed out that some adolescents are at times perpetrators of mob justice:

If a boda boda person (motorbike taxi driver) is knocked down whether he is drunk or not, they call their colleagues … they follow the vehicle and when they get it, they will beat him up (the driver) and kill him. (Male young adult)

##### Social events

Social events, mainly funeral events (nighttime “disco matanga”) and disco parties, were considerably discussed (by half of the KIIs and eight FGDs) as common events during which adolescents perpetrate and fall victim to unintentional injury and violence. During such events, the occurrence of excessive substance abuse and sexual and physical violence is reported as common.

#### Public policy level

##### Social media influence

The influence of watching violence and crime in videos or other social media platforms as well as cyberbullying was discussed as some of the mechanisms through which social media predisposes adolescents to violence and unintentional injury:

… you may meet someone in Facebook … The person may turn not who you expected and he may force you to have sex. (Female secondary school student).

### Underlying protective factors for unintentional injury, violence, and self-harm and suicide-related behavior of adolescents

The protective factors were only discussed at interpersonal and broader ecological levels of community and public policy. With the exception of two factors, all the protective factors were shared across the three categories of injury and violence.

#### Interpersonal level

##### Parental monitoring

This was discussed in the context that some caretakers proactively find out about their adolescents’ whereabouts; restrict their children’s movements during potentially risky conditions like late night hours; and that they provide care, support, and advice. These forms of responsibilities of caretakers were perceived to mitigate against injury, violence, and self-harm or suicidal behavior.

#### Community level

##### Attendance of school

Being in attendance of school, especially with existing school guidance and counseling programs, and well-implemented school rules and regulations, was discussed as a significant protective factor in six of the KIIs. School curricula were also perceived as beneficial because they include some subjects and general messages on prevention of injury, violence, self-injury, and suicide.

#### Public policy level

##### Legal framework

This was discussed as an important protective factor against violence during two FGDs and KIIs. Local police officers and county chiefs were reported to intervene during many cases of crime such as theft and sexual violence. A key informant also mentioned that legal regulations had been proposed to ban funeral parties in Kilifi owing to their associated consequences like sexual and physical violence.

## Discussion

Our findings on prevalent forms of injury and violence are consistent with the top causes of death among adolescents in Eastern Africa (Kassebaum et al., 2017). Noteworthily, our participants’ views on common occurrence of genital trauma resulting from sexual assault and unsafe abortion are also corroborated by reports of high occurrence of sexual and gender-based violence in Kilifi County (Kesho-Kenya, 2016; Marsha, 2015; NCPD, 2017). Most of the young people’s views on forms of injury and violence and their underlying factors were corroborated by those from stakeholders. However, a few disparities emerged about the perceived occurrence of some forms of injury and violence. Cyberbullying and self-inflicted injury were, for instance, only discussed by young people. Moreover, more discussions on forms of violence like bullying and vandalism; forms of suicidal and self-harm behavior such as attempted poisoning; and genital injury were contributed mostly by young people than stakeholders. These disparities may be potentially explained by the fact that adolescents rarely report or seek care for non-fatal or less serious injuries and forms of violence and therefore the occurrence of such problems may remain poorly recognized by stakeholders within their community. These findings however highlight that adolescents can reliably recognize their health needs and concerns and thus they need to be more meaningfully engaged across all levels of research and health promotion. Similar to views from Hadfield and Haw (2001), young people are usually in the best position to talk about being young and can share their experiences with professionals in unique ways.

We found that there are some specific overlapping intra- and interpersonal risk factors which were common for all the three forms of injury and violence. The intrapersonal risk factors included gender, drug use, and poor coping strategy to socio-emotional difficulty. Indeed, drug use (alcohol and other psychoactive drugs) by adolescents is an extensively documented risk factor globally (Schulte and Hser, 2013); moreover, it has also been found that at times drug use is perceived as a way of coping with socio-emotional difficulty by adolescents (Lyness and Koehler, 2016). Similar to our findings on socio-emotional difficulty, coping with romantic relationship concerns has been linked to an increase in risk for suicidal and violent behavior of adolescents (Price et al., 2016). Household poverty and poor caretaker-to-child relationships were interpersonal risk factors reported to predispose adolescents to all the three forms of injury and violence. Some studies have found a close link between poverty-related issues (like despair and depression) and inadequate parenting behavior which demonstrates an overlap between the two factors (Russell et al., 2008). In corroboration with our findings, an inverse relationship between positive parenting practices (like parental monitoring, support, and positive relationship) and adolescents’ aggressive behavior such as weapon carrying and physical fights has been documented (Orpinas et al., 1999).

An overlap of risk factors across different forms of injury and violent behavior suggests that adolescents who experience these shared cumulative risk factors are more likely to face multiple or co-occurring forms of injury and violence. Such a tendency for co-occurrence of injury and violence behavior like self-harm, bullying perpetration, and victimization has been reported among other adolescent sub-populations (Heerde and Hemphill, 2018). Besides, the finding on overlapping risk factors also suggests that the action taken to address/tackle any or some of these shared risk factors is likely to have a preventive spillover effect across various forms of injury and violence behavior.

There was more diversity across socio-ecological levels regarding risk factors shared between unintentional injury and violence. School drop-out was a major intrapersonal-level factor which potentially influences adolescents’ engagement in risky economic activities and problem behavior. It also lessens the protective effects of strict behavioral monitoring by school authorities. Similar consequences of school drop-out have been reported in many low-resource settings such as Bangladesh, Nigeria, Ghana, and Vietnam (Putnick and Bornstein, 2015). We also found that sports activities contribute to both unintentional injury and violence, and our findings are corroborated by a recent study which estimates that over 23 million African adolescents sustain sports-related injuries annually (LeBrun et al., 2018). Our findings showed that peer pressure as an interpersonal-level factor relates to broader community-level risk factors like adolescents’ engagement in mob justice and hazard-prone social events. Similar findings on peer pressure and Kenyan adolescents’ engagement in hazard-prone social events like disco funerals have been reported in other studies within the Kenyan context (Njue et al., 2009).

In connection to specific behavior, our findings on risk factors for unintentional injury showed that specific intrapersonal risks are intertwined and emerge under certain prevailing circumstances within the broader community context. For instance, the influx of motorbike taxis in Kilifi and certain sources of livelihood like stone quarrying, alcohol brewing, and fishing coincided with reports of adolescents’ intrapersonal risk factors like recklessness traffic behavior and underage or inexperienced operation of motorbike taxis. Risks of injuries like cuts, bruises, and falls were also attributed to adolescents’ engagement in activities like manual labor and stone quarrying. Similar to this finding, the occurrence of occupational injuries among adolescents is widely documented and the socio-economically marginalized are often disproportionately impacted (Rauscher and Myers, 2008).

Our findings suggest that social identities such as gender and masculinity as well as social and cultural norms are important factors which can shape the context in which injury and violence occur during adolescence. As an example, in this study, adolescent girls compared to their male counterparts were reported to experience greater risk for genital injury and sexual violence, whereas their male counterparts were perceived as more vulnerable to perpetration of violence and falling victim to unintentional injury, especially those attributable to sensation seeking and display of masculinity. Social events like funeral events which are also engrained in traditional norms were identified as sources of risk for injury and violence. Indeed, some studies emphasize that opportunities and rewards for violence can differ for women and men, and that more often women may suffer longer term physical, psychological, and economic burden resulting from violence, abuse, and injury (Aisenberg et al., 2010; White, 2009). Therefore, public policy and efforts to prevent injury and violence need to take into consideration the existing effects of gender, social identity, and cultural norms in shaping injury and violence for both the perpetrators and victims.

Furthermore, our findings show that violence is momentously attributed to insecurity concerns cutting across the household and broader community context. Similar issues of weapon carrying (Pickett et al., 2005), crime perpetration by close family members (Lalor, 2004), and concerns of neighborhood insecurity, especially in socially deprived communities (Kruger et al., 2007), are shared by adolescents from other parts of the world. In Kilifi, locally appropriate interventions to tackle insecurity such as community policing (“Nyumba Kumi” initiative) (Kioko, 2017) are existent; however, their implementation needs to be strengthened.

Our findings also indicate that poor adolescent mental and psychological health is a recognized risk factor for self-harm and suicidal behavior of adolescents in Kilifi setting. Similar to these findings, some research on suicidal behavior of adolescents within a Kenyan context showed that co-occurrence of psychiatric disorders greatly increased the risk for suicidal behavior and that the risk was higher among older adolescents (16–18 years) compared to their younger counterparts (Khasakhala et al., 2013).

Our results on protective factors against adolescents’ injury, violence, and self-harm or suicidal behavior highlight two important issues. First, some specific protective factors like good parenting style and school attendance can potentially address the risk for multiple forms of injury and violence. In line with this interpretation, evidence on adolescent injury prevention recommends that education and skills development for adolescents should be integrated with parental/family-based components to form an injury prevention approach which tackles multiple forms of injury or violence in preference to standalone measures (Harvey et al., 2009). Second, although views on protective factors were fewer than those on risk factors, there was still an indication of the diversity in socio-ecological levels of protection, that is, that protective factors for a specific form of injury or violence are often generated from different levels of human interaction. Coherent with our observation, a comprehensive approach to injury prevention identifies six inter-related levels comprising individual-oriented actions (strengthening individual knowledge and skills), community-oriented actions (community education, fostering coalitions, and networks), organization-related actions (changing organizational practices, educating service providers), and influencing policy and legislation (Cohen and Swift, 1999).

Drawing from our results, we identify some suggestive actions (which may be part of a multi-component intervention) to address underlying causes of injury, violence, and self-harm behavior of adolescents in Kilifi. We propose that adolescents are targeted with tailored life skills education so as to build specific individual competences like problem solving and critical thinking that help curtail injury and violence behavior (WHO, 2009). Subsidized education (e.g. subsidized vocational training programs (Attanasio et al., 2011)) to empower youth and build transferable skills is important and should be backed by safer youth employment opportunities and policies. School- and community-based interventions which engage multiple stakeholders to build safer communities and challenge suppressive social norms (like those that condone gender-based violence) are potentially feasible solutions for intimate partner and sexual violence (Lundgren and Amin, 2015). Behavioral family interventions promoting good parenting skills and knowledge on injury prevention are crucial; however, they need to incorporate income-generating skills to address overarching family-level poverty. There is also a need for better enforcement of policy-level strategies to regulate poor operation of public transportation and to enforce strict traffic regulations as has been emphasized in other studies across SSA (Ajay, 2011). Finally, mental health promotion is another crucial element that should be incorporated and prioritized in injury prevention programs. Nonetheless, all the above actions are only suggestive.

Future research is urgently needed to quantify the prevailing burden of injury, violence, and self-harm or suicidal behavior of adolescents in Kilifi. Furthermore, there is an urgent need for research to pilot and test the applicability and effectiveness of interventions tackling underlying determinants of injury and violence of adolescents in this setting.

### Strengths and limitations

A major strength of this work is that participants comprised a diverse group of key informants (such as clinicians, teachers, and social workers) and young people like primary and secondary school students, adolescents who had dropped out of school, and young adults. This ensured that the views were diverse, triangulated, and contrasted across different study participant groups. We also explored the different forms of injury and violence-related behavior using a socio-ecological approach and thereby obtained clearer insight of the complexity of the forms and underlying factors for violence and injury of adolescents in Kilifi. However, our findings are from a rural setting and circumstances may differ within urban settings. We may also not completely rule out the possibility that some of the descriptions of the participants might not reflect the actual extent of the problem, for instance, some rare incidents such as self-inflicted body injuries, snake bites, and mob justice described during a few FGDs and KIIs. Therefore, our findings need to be interpreted with caution.

## Conclusion

The young people and local stakeholders’ views indicate that unintentional injuries, especially road traffic accidents, falls, and drowning, interpersonal violence, especially fights and gender-based violence, and forms of self-injury or suicidal behavior are of concern among adolescents in Kilifi. Many of the underlying risk factors overlap. In order to address injury and violence among adolescents in Kilifi, we recommend multi-component interventions targeting more than one form of injury and violence. There is so far scanty evidence of feasible interventions to address injury and violence of adolescents in Kilifi. Nonetheless, we present some suggestive actions based on our research findings. We recommend for life skills education, behavioral family approaches incorporating income-generating activities, initiatives to improve school retention and development of transferrable skills, socially mobilizing communities for collective responsibility, and mental health promotion. From a broader perspective, stronger enforcement of youth responsive policies to address unemployment and to combat injury and violence is timely. There is an urgent need for research to quantify the burden of injury and violence as well as to pilot and test feasibility of injury prevention interventions in this setting.
